# Supplementary material for: Complexome profiling of the Chlamydomonas psb28 mutant reveals TEF5 as an early PSII assembly factor
Source: Plant Cell. 2025 Mar 17;37(6):koaf055. doi: 10.1093/plcell/koaf055 (PMC12164590; doi:10.1093/plcell/koaf055)
Supplement: koaf055_Supplementary_Data [file koaf055_supplementary_data.zip › psb28_interactive.zip!/plots/intensity/qualityPlots/peptides/rps7_Cre-1.g2716973.t1.p1.html]

### Cre-1.g2716973.t1.p1

Trivial name: rps7  
SeqCoverage [median]: 5.40  
Mass: 19.13 kDa  
  
 

| Sequence | MissedCleavage | Mass [Da] | wtIntensity | muIntensity | Score |
| --- | --- | --- | --- | --- | --- |
| AGAIQMVPR | 0 | 941.51 | 363.49 | 1723.74 | 129.40 |
|  |  |  | 363.5 | 1723.7 |  |
